# Acquired Resistance to Osimertinib in *EGFR*-Mutated Non-Small Cell Lung Cancer: How Do We Overcome It?

**DOI:** 10.3390/ijms23136936

**Published:** 2022-06-22

**Authors:** Elisa Bertoli, Elisa De Carlo, Alessandro Del Conte, Brigida Stanzione, Alberto Revelant, Kelly Fassetta, Michele Spina, Alessandra Bearz

**Affiliations:** 1 Dipartimento di Oncologia Medica, Centro di Riferimento Oncologico di Aviano (CRO) IRCCS, 33081 Aviano, Italy; elisa.bertoli@cro.it (E.B.); elisa.decarlo@cro.it (E.D.C.); alessandro.delconte@cro.it (A.D.C.); brigida.stanzione@cro.it (B.S.); kelly.fassetta@cro.it (K.F.); michele.spina@cro.it (M.S.); 2 Department of Medicine (DAME), University of Udine, 33100 Udine, Italy; 3 Dipartimento di Radioterapia, Centro di Riferimento Oncologico di Aviano (CRO) IRCCS, 33081 Aviano, Italy; alberto.revelant@cro.it

**Keywords:** osimertinib, acquired resistance, *EGFR*-mutated, NSCLC

## Abstract

Osimertinib is currently the preferred first-line therapy in patients with non-small cell lung cancer (NSCLC) with common epidermal growth factor receptor (*EGFR*) mutation and the standard second-line therapy in T790M-positive patients in progression to previous EGFR tyrosine kinase inhibitor. Osimertinib is a highly effective treatment that shows a high response rate and long-lasting disease control. However, a resistance to the treatment inevitably develops among patients. Understanding the secondary mechanisms of resistance and the possible therapeutic options available is crucial to define the best management of patients in progression to osimertinib. We provide a comprehensive review of the emerging molecular resistance mechanism in EGFR-mutated NSCLC pre-treated with osimertinib and its future treatment applications.

## 1. Introduction

To date, the treatment of non-small-cell lung cancer (NSCLC) is strictly related to molecular profiling, which has allowed the development of target therapies that have changed the natural history of this disease in recent decades. The Epidermal Growth Factor Receptor (*EGFR*)-activating mutations are the most common molecular aberrations in NSCLC, being present in 15% of the Caucasian population and up to 50% of the Asian population with advanced NSCLC [1,2]. Exon 19 deletion or L858R point mutation in exon 21, also known as “common mutation”, account for about 90% of these mutations [2]. EGFR tyrosine kinase inhibitors (TKIs) have been demonstrated to be superior over classical chemotherapy in this population and represent the standard of care for *EGFR*-mutated NSCLC [3,4,5]. The secondary mutation of T790M is the most commonly acquired resistance mechanism to first- and second-generation EGFR TKIs and it develops in over half of patients [6,7,8]. T790M is a gatekeeper mutation that increases the affinity for ATP in the ATP-binding domain of *EGFR*. As a result, it reduces the potency of first- and second-generation EGFR TKIs that have an ATP-competitive mechanism of action [9]. To overcome this resistance mechanism, third-generation TKI have been developed. Osimertinib is the most used third-generation TKI designed to target both *EGFR*-sensitising mutations and T790M in a selective way [10]. Originally, osimertinib was approved only in *EGFR* T790M-positive mutation NSCLC patients progressing following EGFR TKI therapy [8]. Later, the FLAURA study demonstrated a significant improvement in progression-free survival (PFS) and overall survival (OS) compared to treatment with first-generation EGFR TKIs [11]. Additionally, an improved tolerability profile (due to high selectivity against mutant vs. wild-type *EGFR*) and substantial central nervous system activity made osimertinib a widely adopted treatment even in the first-line setting, regardless of whether T790M is present [11,12].

Unfortunately, patients inescapably develop secondary resistance, which constitutes a critical challenge due to the scarcity of post-osimertinib pharmacological options available. The resistance mechanisms to a third-generation TKI are complex and not fully understood, with some differences depending on whether osimertinib is the first- or second-line treatment [13,14]. Understanding the resistance mechanisms to osimertinib and the possible treatment options available is essential in choosing the subsequent therapy strategy, largely because of upfront setting anticipation of osimertinib. The purpose of this review is to give a usable overview of our current knowledge about the emerging resistance mechanisms to osimertinib in patients with *EGFR*-mutated NSCLC and relevant therapeutic options.

## 2. Acquired Resistance to Osimertinib

The acquired resistance mechanisms to osimertinib may be either dependent (-“on target”) or independent (-“off-target”) of the *EGFR*. In the first case, tumour cell proliferation remains directly linked to EGFR signalling. In off-target resistance, other parallel molecular pathways circumvent EGFR signalling. The resistance mechanisms to osimertinib appear to be analogous in the first- and second-line settings [15]. Nevertheless, the off-target mechanism of resistance may be more relevant in first-line osimertinib than in later-line treatment, in which tumour cells have previously displayed dependence on *EGFR* through T790M mutation [16].

The plasma analysis of circulating tumour DNA (ctDNA) by NGS in the FLAURA and AURA3 studies in patients with progressive disease during osimertinib therapy provided us the majority of available data [13,14].

Table 1 summarises the ongoing clinical trial in pretreated *EGFR*-mutant metastatic NSCLC.

**Figure 1:**
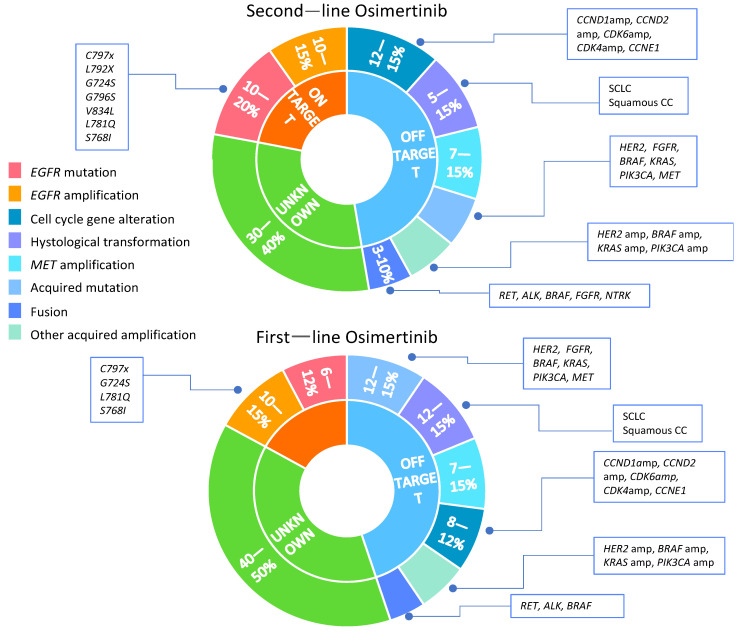
Acquired resistance mechanism to osimertinib in first- and second-line treatment. Amp—amplification; SCLC—small cell lung cancer; squamous CC—squamous cell carcinoma.

Figure 1 summarises the acquired resistance mechanism to osimertinib in first- and second-line treatment.

### 2.1. EGFR On-Target Alterations

#### 2.1.1. T790M Loss

In 50–60% of patients treated with first- or second-generation TKI, the somatic mutation of resistance p.Thr790Met (T790M) develops, resulting from a gatekeeper mutation in exon20 of *EGFR* [17]. T790M causes steric hindering of the binding to their connected ATP-binding site on EGFR of an ATP-competitive kinase inhibitor (first- and second-generation TKI), but irreversible inhibitors (third-generation TKIs) overcome this resistance simply through covalent binding [9]. More precisely, osimertinib irreversibly and covalently binds the cysteine-797 residue in the ATP binding pocket of EGFR, regardless the hindering of T790M. Furthermore, since osimertinib creates an irreversible link with the ATP pocket of the EGFR it is able to overcome the increased affinity of ATP determined by the T790M mutation [10]. Data from the AURA3 trial highlight that about 50% of patients who received second-line osimertinib (and 100% of whom developed a dependent *EGFR* tertiary mutation) at the time of progression preserved the T790M mutation [13]. In the remaining cases, resistance to osimertinib commonly showed the loss of T790M and was frequently associated with the development of *KRAS* mutation, gene fusions, histological transformation and other rarer mechanisms [13,18]. Interestingly, earlier resistance and poorer survival was associated with T790M loss [18,19]. As expected, at progression to front-line osimertinib treatment no evidence of T790M mutation occurred [14] and, given the anticipation of osimertinib in the first-line setting, the incidence of T790M is likely to become less and less relevant.

#### 2.1.2. C797x

In *EGFR*m/T790M patients, second-line treatment with osimertinib can lead to the emergence of the so-called EGFR “triple mutant”. The most common tertiary *EGFR* mutation is the point mutation C797 in exon 20 and it accounts for 15–26% of cases of resistance to second-line osimertinib treatment [11,13,20]. The most commonly found substituted amino acid is serine (C797S), and glycine (C797G) has been anecdotally reported [20,21]. Indeed, the bond to the residue C797 in the ATP pocket is how osimertinib exceeds the T790M resistance [22,23]. Moreover, it is the second most recurrent resistance mechanism (7%) after *MET* amplification in first-line osimertinib [14]. Case reports have described that in the absence of T790M mutation, cancer cells harbouring C797S mutation maintain first- and second-generation EGFR TKI sensitivity [24]. The same is true in cases where C797S mutation coexists in a different allele with T790M (trans)—if the C797S mutation is in the same allele (cis), as in most T790M mutated cases, sensitivity to first and second-generation TKI is lost [22,25]. Two case reports reported patients with EGFR C797S mutation located in trans with T790M who experienced an initial response with the association of a first-generation TKIs and osimertinib [25,26]. Additionally, in vitro and in vivo activity of fourth-generation EGFR TKIs, alone or in combination with osimertinib, have been demonstrated. These new generation of EGFR TKIs such as EAI045, JBJ-04-125-02 and BLU-945, overcome both T790M and C797S mutations. However, they still have not been assessed in a clinical trial [27,28,29]. Moreover, the addition of the ALK inhibitor brigatinib to a fourth-generation EGFR TKI has revealed in vivo activity in triple-mutant EGFR/T790M/C797S [30]. Interestingly, amivantamab (a bispecific anti-EGFR and anti-MET inhibitor) showed response in patients with coexisting C797S mutation and MET amplification [31] and brigatinib plus cetuximab may be an efficacious therapy option in patients with T790M/cisC797S mutations resistant to osimertinib [32]. BDTX-1535, an orally available, highly potent, selective, irreversible inhibitor of allosteric EGFR alterations (NCT05256290), BLU-945, a selective EGFR inhibitor, as monotherapy or in combination with osimertinib [NCT04862780], BLU-701 in monotherapy or in combination with bot osimertinib or platinum-based chemotherapy (NCT05153408) are currently in testing in phase I/II clinical trials (Table 1).

#### 2.1.3. Other EGFR Tertiary Mutations

*EXON 18*. Mutation in L718Q/V residue interacts directly with osimertinib in the EGFR kinase domain of the ATP binding site [33]. Of note, NSCLC with EGFR L858R/T790M/L718Q/V mutation are resistant to all EGFR-TKIs but L858R/L718Q/V (commonly found in patients who develop resistance to osimertinib [34]) seems to retain sensibility to afatinib [35]. Furthermore, the rare G724S mutation has been outlined as a resistance mechanism to second-line osimertinib [36]. In a subgroup of EGFR T790M negative but G724S mutated and osimertinib-resistant patients, Fassunke et al. demonstrated in vitro that afatinib reduces tumour growth of G724S driven cells [37].

*EXON 20*. G796R/D and L792 mutations were anecdotally reported in NSCLC treated with osimertinib and sterically hindered it, but drug sensitiveness against these newly on-target resistance mechanisms mandates additional investigations [36]. In vitro, double mutant T790M/M766Q are resistant to osimertinib but sensitive to neratinib and poziotinib (dual inhibitors of the human epidermal growth factor receptor 2 (HER2) and EGFR kinase) [38].

### 2.2. EGFR Off-Target Alteration

#### 2.2.1. MET Amplification

*MET* amplification is one of the most common mechanisms of acquired resistance to osimertinib, with a prevalence of 19% and 15% of patients, respectively, receiving second- and first-line therapy [13,14,18]. *MET* amplification bypasses EGFR by causing persistent activation of downstream signalling paths mediated by phospho-inositide 3-kinase (PIK3CA), mitogen-activated protein kinase (MAPK), and signal transducer and activator of transcription (STAT) [39]. At present, *MET* amplification is generally defined as the presence of a *MET* gene copy number of ≥5 or a *MET/CEP7* ratio of ≥2 [18]. So far, there is an absence of agreement on the definition of *MET* amplification detected by next-generation sequencing (NGS) in liquid biopsy, and NGS or fluorescence in situ hybridisation (FISH) to detect *MET* amplification should be carried out on all biopsies performed to define osimertinib resistance. NGS allows the parallel identification of single-nucleotide variants, rearrangements, deletions, insertions, copy number variations, and the definition of distinct thresholds [21,40]. However, not all NGS-based assays control for *CEP7*; consequently, a detected increase in copy number may actually be a polysomy instead of a proper *MET* amplification. [41]. Therefore, FISH is advised if NGS does not expressly assess for gene copy number gain [42]. *MET* amplification may emerge with or without T790M loss in the second-line osimertinib setting. In 7% of cases, it co-occurs with the tertiary mutation EGFR C797S [13] and is also potentially associated with *CDK6* and *BRAF* amplification [43]. Preclinical evidence shows the osimertinib resistance in *EGFR*-mutated cell lines with *MET* amplification could be overcome by the concomitant use of MET inhibitors with afatinib [44]. Case reports suggested that combining crizotinib (a TKI with dual anti *ALK* and *MET* activity) with osimertinib or erlotinib might get over MET-mediated resistance [45,46,47]. Various combinations of EGFR and MET TKI are currently under investigation. Recently, data have been published from an interim analysis of the phase study Ib TATTON, which investigates the combination osimertinib-savolitinib, a MET TKI, in patients with *MET* amplification defined as *MET* gene copy number ≥ 5 or *MET:CEPT7* ≥ 2. An objective response rate (ORR) of 30% and a PFS of 5.4 months were obtained in third-generation EGFR TKI pretreated patients, reaching 64–67% and 9–11 months, respectively, in third-generation EGFR TKI-naïve patients based on the different cohorts analyzed [48]. Phase II trials with this combination are currently ongoing (NCT03778229 (SAVANNAH), NCT03944772 (ORCHARD)). Two phase I/II studies tested the combination of the first-generation gefitinib with a MET inhibitor. The association of capmatinib with gefitinib obtained an ORR of 27% and an increased ORR of 47% was seen in patients with MET gene copy number ≥ 6 [49]. Similarly, the combination of tepotinib with gefitinib resulted in longer PFS and OS in compared to chemotherapy in the INSIGHT trial [50]. Initial results from the CHRYSALIS phase I trial, still ongoing, showed an ORR of 36% and a mPFS of 4.9 months in the osimertinib-resistant cohort was described with amivantamab combined with lazertinib (third-generation EGFR TKI, brain-penetrant) [51]. At the recent ASCO 2022 Annual meeting, updated results of pretreated cohort has been presented. At a median follow up of about 8 months, an ORR of 36% was confirmed in patients pretreated with first- or second-line osimertinib, with a clinical benefit rate of 58% and a median duration of response (mDoR) not reached. Moreover, in patients heavily pretreated with at least osimertinib plus a platinum-based chemotherapy, an ORR of 29% with a mDoR of 8.6 months was described. A manageable safety profile was confirmed [52].

Telisotuzumab vedotin is a MET-directed antibody–drug conjugate (ADC) that is in testing in a currently ongoing phase 1/1b study NCT02099058 in monotherapy or combination with osimertinib, erlotinib or nivolumab in patient harboring cMET overexpression after prior osimertinib therapy. In the interim analysis, the encouraging results of an ORR of 58% observed for the combination of telisotuzumab vedotin with osimertinib and an acceptable safety profile were presented at the ASCO 2022 annual meeting [53].

Other ongoing clinical trials targeting MET amplification are listed in Table 1.

#### 2.2.2. HER2 Amplification

Another off-target mechanism that bypasses EGFR through the activation of downstream PI3K–Akt and MAPK/pathways is the overexpression of ErbB2, a tyrosine kinase receptor encoded by the *HER2* gene. In patients who developed resistance to second-line and first-line osimertinib, *HER2* amplification was detected in 5% and 2% of cases, respectively. Interestingly, *HER2* amplification is mutually exclusive with T790M [13,14]. *HER2* amplification resistance was sensitive to osimertinib plus the ADC anti-HER2 trastuzumab-emtansine (TDM1) in preclinical models. Recently, Li et al. demonstrated activity of TDM1 even in patients with *EGFR*-mutated *HER2* amplification NSCLC who experienced disease progression on previous EGFR TKI [54]. Moreover, they described that ADC switching from TDM1 to trastuzumab deruxtecan (T-DXd), holding a distinct cytotoxic payload, achieves durable responses in a NSCLC patient that developed resistance to T-DM1 [54]. The TRAEMOS phase I/II trial is investigating the osimertinib–TDM1 combination in patients who progressed to an EGFR TKI gaining *HER2* amplification (NCT03784599).

#### 2.2.3. RAS/MAPK Pathway Mutations

*KRAS* mutation or amplification and *NRAS*, *MEK1* and *BRAF* mutation have all been reported as acquired resistance to osimertinib [39]. In the FLAURA trial, variable mutations of *NRAS* (e.g., E63K mutation) and *KRAS* (G12S, G13D, Q61R, Q61K, G12D mutations) were discovered in 3% and 1% of patients who progressed on first-line and second-line treatment, respectively [14,43]. *BRAF* V600E mutation was found in about 3% of cases, with or without T790M, both in first- and second-line osimertinib [13,14,18]. Furthermore, *BRAF* V600E mutation coexisting with *MET* amplification as resistance mechanisms to first-line osimertinib therapy was reported [55]. *BRAF* V600E-mutated cell lines after osimertinib treatment showed sensitivity to combining a BRAF inhibitor (encorafenib) and osimertinib [56]. Likewise, Xie et al. proved that osimertinib combined with vemurafenib (a BRAF inhibitor) effectively overcame *BRAF* V600E-mediated osimertinib resistance [57]. Furthermore, both in vitro and in vivo, salumetinib (a MEK inhibitor) combined with osimertinib has been proven to overcome TKI resistance caused by *NRAS* mutations, although further evidence of this combination is required [58]. The association of dabrafenib and trametinib in *BRAF* V600E mutated NSCLC, including *EGFR*-mutated patients who progressed to an EGFR TKI is currently under investigation (NCT04452877).

#### 2.2.4. PI3K Pathway Mutations

Activation of PI3K, either via *PIK3CA* mutations (*E454K*, *E542K*, *R88Q*, *N345K*, *E418K*) or *PTEN* deletion, is involved in 4–11% of patients who progressed to osimertinib [18]. Because of *PIK3CA* has a role in several oncogenic pathways in NSCLC, in contrast to other oncogenic driver mutations which are generally mutually exclusive, PIK3CA mutation is frequently contextual to other oncogenic gene mutations [59]. In patients with associated *PIK3CA* and *EGFR* mutations treated with EGFR TKI monotherapy no significant dissimilarities in clinical results were recorded [59]. To our knowledge, no targeted therapy against *PIK3CA* mutation has demonstrated clinical benefit thus far.

#### 2.2.5. Oncogenic Fusions: FGFR3, RET, NTRK

Chromosomal rearrangements involving driver oncogenes, namely the oncogenic fusions, have been identified mainly in second-line osimertinib resistance (4–7%) [13,18]. Oncogenic fusions include but are not limited to *ALK* (*SBTBN1-ALK*, only in first-line osimertinib resistance, *PLEKHA7-ALK*), *BRAF* (*AGK-BRAF*, *PCBP2-BRAF*, *ESYT2-BRAF*, *BAIAP2L1-BRAF*), *FGFR* (*FGFR3-TACC3*), *NTRK* (*NTRK-TMP3*), *RET* (*RET-ERC1*, *CCD&-RET*, *NCOA4-RET*) and *ROS1* (*GOPC-ROS1*) [13,14,18,60]. Zeng et al. reported that in a *GOPC-ROS1* rearranged patients, a combination of crizotinib and osimertinib was proven effective and well tolerated [60]. Piotrowska et al. published the experience of two patients with acquired *CCDC6-RET* fusion who had rapid responses to the combination of osimertinib–RET inhibitor (BLU-667) [61]. Moreover, in a series of 12 patients and in a case report with *RET* fusions as mechanism of osimertinib resistance the combination of osimertinib and the RET inhibitor selpercatinib was feasible and achieved radiological response [62,63]. One patient in progression to osimertinib who developed *PLEKHA7-ALK* fusion obtained a durable response with the addition of alectinib (an ALK TKI) to osimertinib. The combination therapy targeting EGFR and the acquired fusion achieved clinical benefits in numerous patients [64]. Another brief report showed that in two patients with *ALK-AML4* fusion, osimertinib association with both crizotinib and alectinib obtained disease control [65]. Although the data come from case reports or small series, they are encouraging to think about in the development of target therapy combinations even in this setting.

#### 2.2.6. Cell Cycle Alterations

Plasma analysis of the studies AURA3 and FLAURA found that 10% of resistance mutations in first-line osimertinib and 12% in second-line treatment are represented by alteration of cell cycle-related genes. These include amplification or mutations in cyclin D1/2 and *E1* genes, cyclin-dependent kinase (CDK) 4/6 and CDK inhibitor 2A genes [13,14]. The combination of CDK4/6 inhibitor palbociclib and osimertinib overcame the acquired resistance of osimertinib in cell lines [66]. Analogously, La Monica et al. provided preclinical evidence for employing abemaciclib (monotherapy or in addition to osimertinib) to overcome resistance in patients progressing to first-line osimertinib. They also suggested the combination of osimertinib and abemaciclib as a potential approach to prevent or delay resistance to osimertinib in first-line therapy [67]. Ongoing clinical trials are listed in Table 1.

#### 2.2.7. Other Mechanisms

Anexelekto (AXL) belongs to the receptor tyrosine kinase family, implicated in cell proliferation, survival and migration. Upregulated AXL interacting with EGFR and HER3 induces intrinsic and acquired osimertinib resistance [68]. In cell lines, the combination of osimertinib and cabozantinib has been reported to overcome osimertinib resistance [68]. Moreover, several authors have suggested the preclinical efficiency of using AXL inhibitors combined with osimertinib on cell lines resistant to osimertinib, making it an attractive pharmacological target [69,70,71].

Aberrant activation of the insulin-like growth factor 1 receptor (IGFR1R) has been suggested as one non-genetic cause of third-generation EGFR TKI resistance in T790M mutated NSCLC [72]. Adding an IGFR1 inhibitor to osimertinib might efficaciously overcome the acquired resistance to osimertinib elicited by IGF1R activation [73].

Patritumumab deruxtecan is an ADC directed against HER3 (*ErBB3*), another often-overexpressed receptor in *EGFR*-mutated NSCLC. HER3 alterations do not directly mediate resistance to EGFR-TKIs, but HER3 activates oncogenic signalling pathways, including PI3K and MAPK. Nevertheless, patritumab deruxtecan, in a phase I clinical trials in osimertinib-resistant patients, achieved a response rate of 39%, irrespective of the underlying resistance mechanism. HER3-directed ADC might provide a future agnostic treatment alternative for the TKI resistance mechanism of EGFR [74]. A prospective clinical trial is currently ongoing, testing patrimumab deruxtecan in combination with osimertinib in patients progressing to first-line osimertinib (NCT04676477).

#### 2.2.8. Histological Transformation

Unlike mutational gene status, a study of tissue samples/re-biopsy is required to assess the existence of histologic transformation as an acquired resistance mechanism to osimertinib. The histologic conversion from *EGFR*-mutated NSCLC into small cell lung cancer (SCLC) has been recorded in 14% of patients progressing to first-line osimertinib and in 4–15% of patients experiencing disease progression in the second-line setting [6,18,61]. Notably, the risk of SCLC transformation has been significantly associated with the contemporary presence of *RB1* and *TP53* mutations, while no SCLC cases were recorded in wild-type patients [75,76]. Therefore, for lack of other resistance mechanisms, a liquid biopsy positive for *RB1* or *TP53* alterations may imply that a tissue re-biopsy should be considered to search for SCLC transformation. Likewise, in about 15% of patients receiving both first- and second-line osimertinib, squamous cell transformation is described [77]. Both in SCLC and squamous transformation, the original *EGFR*-mutation is retained [75,78]. Currently, for *EGFR*-mutant NSCLC with transformed histology, which generally has a worse prognosis as a consequence of intrinsic resistance mechanisms, there are no target therapies or therapeutic strategies validated [75,76]. Histology-driven chemotherapy would remain the standard of care in this patient subgroup even if traditional systemic chemotherapy yielded limited efficacy, notwithstanding some evidence of efficacy of platinum-etoposide chemotherapy—but no immune checkpoint inhibitors (ICIs)—in SCLC-transformed NSCLC [75,76,79]. Of note, the association of a PARP inhibitor (niraparib) and anti PDL1 durvalumab is currently under investigation in SCLC-transformed *EGFR*-mutated NSCLC (NCT04538378).

Resistance to osimertinib has also been described in epithelial-to-mesenchymal transition (EMT) and over-expression of its transcription factor TWIST-1 by NSCLC cells. The assumption of a mesenchymal phenotype confers migratory capacity to the cells through the loss of the expression of cadherin in favor of vimentin [80]. In the preclinical setting, TWIST-1 inhibitors are under investigation [81].

## 3. New First-Line Combinations Aming to Prevent the Onset of Resistance

Because in up to 40–50% of cases, there are currently no known detectable changes and as osimertinib is the first-line choice TKI and chemotherapy is the second standard line, the researchers are studying the possibility of preventing the emergence of resistance to third-generation EGFR TKI by combining, in various ways, TKI, chemotherapy, and immunotherapy in the front-line setting. Ongoing clinical trials in this setting are listed in Table 2. 

### 3.1. Chemotherapy

Clinical data support the superiority in terms of PFS, of the combination of a first-generation TKI and chemotherapy versus EGFR TKI monotherapy [82,83,84]. Moreover, the osimertinib–chemotherapy combination had a good safety profile and demonstrated promising control of central nervous system (CNS) disease in a group of patients who progressed systemically to at least two lines of therapy, including an EGFR TKI [85]. The ongoing phase III trial FLAURA2 compares first-line osimertinib plus a platinum-pemetrexed based chemotherapy with osimertinib alone in *EGFR*-mutated NSCLC. The first results published encouragingly demonstrated manageable safety and tolerability of this combination [86]. Additionally, a study designed to prevent SCLC transformation is combining platinum–etoposide chemotherapy plus osimertinib in the first-line setting for *EGFR*-mutated NSCLC with concurrent *RB1* and *TP53* mutations (NCT03567642).

### 3.2. VEGF Inhibitors

Another association of interest is between EGFR TKI and an antiangiogenic agent. The rationale is that VEGF signalling is regulated by EGFR expression and shares common downstream pathways; conversely, an EGFR independent VEGF up-regulation is supposed to promote resistance to EGFR inhibition. [87]. Even if the exact mechanism is currently not well understood, there is preclinical evidence that VEGF/VEGF receptor inhibition boosts EGFR TKI activity [88]. Various trials demonstrate a significant PFS benefit, but not translated into OS advantage, with first-generation EGFR TKI and the anti-VEGF monoclonal antibody bevacizumab or the monoclonal antibody targeting VEGF receptor 2 ramucirumab [89,90,91]. To date, the association in the second-line of bevacizumab and osimertinib in T790M patients failed to show prolongation of PFS vs. osimertinib alone [92], but other clinical trials are ongoing in the first-line setting (Table 1). Nishio et al. recently reported data from the RELAY+ phase III trial in which ramucirumab plus gefitinib achieved a positive 1-year PFS rate with a manageable safety profile in Asian patients in a first-line setting [93]. Data from a concluded phaseI/II study of combination osimertinib and bevacizumab in first-line *EGFR* mutant NSCLC are awaited (NCT02803203).

### 3.3. First-Generation TKI

In the absence of T790M mutation development, the on-target *EGFR* resistance mechanism to osimertinib seems to maintain sensitivity to first- and second-generation EGFR TKI. Consequently, combining a previous generation of TKI with osimertinib could potentially prevent on-target resistance [21]. Concurrent osimertinib plus gefitinib in the first-line setting was safe and obtained an objective response rate consistent with previously reported first-line osimertinib. However, survival outcomes and acquired resistance mechanism results are still awaited [94].

### 3.4. Alternative Pathways Inhibitors

To avoid the emergence of off-target resistance mechanism various strategies trying to co-target *EGFR* and alternative pathways are under investigation in several clinical trials (Table 1). Among these are awaited with particular interest the result of the amivantamab–lazertinib combination. Besides the CHRYSALIS phase I study mentioned above, two phase III clinical trials are currently ongoing. The MARIPOSA study (NCT04487080) compares the efficacy and safety of amivantamab–lazertinib combination therapy versus single-agent osimertinib and the AMIGO-1 trial evaluates the combination of amivantamab–lazertinib with platinum-pemetrexed based chemotherapy in treatment naïve EGFR-mutated NSCLC (NCT05299125).

### 3.5. Immunotherapy

*EGFR*-mutant NSCLC patients have historically been excluded from most first-line trials with immune checkpoint inhibitors. Previous evidence suggested that PD-L1 expression does not predict benefit in *EGFR*-mutant NSCLC [95]. Data from Impower130 showed that the addition of anti-PDL1 atezolizumab to chemotherapy does not improve survival in mutated *EGFR* patients, while from the Impower150 study comes the suggestion that there may be a certain synergy of the anti-VEGF and-PDL1 inhibitors since the addition of bevacizumab to atezolizumab and chemotherapy gave a survival benefit in this population [96,97]. More recently, the final exploratory analysis of Impower150 reported OS benefits for the atezolizumab–bevacizumab–carboplatin–paclitaxel combination against bevacizumab–carboplatin–paclitaxel therapy in patients with sensitising *EGFR* mutations and with liver metastases. Although exploratory, and therefore to be interpreted with caution, these data support a possible use of this combination at the forefront, even if there is no comparison with the standard current therapy with EGFR TKI [98].

No data are currently available in patients with *EGFR*-mutated NSCLC in progression after osimertinib. To elucidate this, the phase III trial KEYNOTE 789 is currently testing the efficacy and safety of pembrolizumab in addition to platinum–pemetrexed-based chemotherapy specifically in *EGFR*-mutated NSCLC in progression after an EGFR TKI, osimertinib included (NCT03515837). The combination of osimertinib with the anti-PDL1 durvalumab investigated in the TATTON study was burdened with high rates of immune-mediated adverse events and, in particular, interstitial lung disease, leading to premature termination of enrollment in this study and in the CAURAL phase III study (NCT02454933) [99]. A meta-analysis of adverse events in combination EGFR TKI and ICIs in advanced *EGFR*-mutant NSCLC confirmed that the joint incidences of gastrointestinal grade 3 skin and adverse events and ILD were significantly higher in combination therapy than in osimertinib monotherapy, limiting future clinical development of this association [100].

## 4. Conclusions

The optimal subsequent treatment after the progression to osimertinib must be tailored according to sites of progression and resistance mechanisms. In patients with limited progression sites, either in the brain or other organs, data support the beyond-progression osimertinib treatment with definitive local therapy, such as stereotactic radiotherapy or surgery of sites of oligo-progression [15,101] and other ongoing clinical trials such as NRG LU002 and SARON are ongoing with the same rationale (NCT03137771, NCT02417662).

In patients requiring a change in systemic therapy, although the standard of treatment is still platinum-based chemotherapy, strategies targeting specific resistance mechanisms are showing promising results, and they should be encouraging access to clinical trials with specific agents targeted at resistance alterations [102].

To date, for assessing osimertinib-resistance mechanisms, the gold standard remains a tissue re-biopsy, which allows histological evaluation, NGS, and an RNA-based fusion panel analysis. If tissue biopsy is not feasible, the ctDNA analysis (liquid biopsy) can be assessed, but it must be taken into account that not all cancers have a ctDNA shedding detectable [18], that histological transformation cannot be detected in liquid biopsy samples [77], and that oncogenic fusions and acquired gene amplifications are not always surely detected by available ctDNA testing. The tissue biopsy should be reconsidered if the ctDNA does not identify the basal *EGFR* mutation or resistance mechanisms. More comprehensive data are awaited from the ELIOS study (NCT03239340). Plasma genotyping and paired tumour biopsy from patients treated with first-line osimertinib will be analyzed by NGS to assess resistance mechanism.

In a first-line osimertinib-relapsed setting, the molecular-driven designed phase II ORCHARD platform trial is testing different agents in combination with osimertinib, according to the identified TKI resistance mechanism. They include, but are not limited to, savolitinib, gefitinib, necitumumab or others in the case of *MET* alteration, C797X mutation, *EGFR* amplification or no biomarker, respectively (NCT03944772). Considering the tropism of the oncogene disease addicted to the diffusion of the brain, the results of the phase 3 study COMPEL (NCT04765059) will be very interesting, evaluating the continuation of osimertinib or placebo with platinum-based chemotherapy in patients with *EGFR*-mutated metastatic NSCLC who responded to first-line osimertinib therapy and subsequently experienced radiological, extracranial disease progression, with stratification based on the presence or absence of specific endpoint encephalic metastases for CNS outcomes. Regardless of the identification of a biomarker, the combination of osimertinib with necitumumab, a highly selective monoclonal antibody against EGFR, was shown to be active and safe in patients with advanced *EGFR*-mutated NSCLC pre-treated with osimertinib in the first-line setting in a phase I study recently presented [103]. Although further studies are warranted, this could be another approach to be explored.

To conclude, after the failure of osimertinib treatment, access to clinical trials, subject to an extensive evaluation of the genomic profile, should be granted to all patients, not only to allow a potential treatment tailored on the basis of the resistance mechanism identified but also to delve deeper into the knowledge of the resistance mechanisms themselves.

## Figures and Tables

**Table 1:** Selection of ongoing clinical trials in osimertinib-pretreated EGFR-mutated advanced NSCLC.

NCT Identifier	Phase	Drug(S) Class	Population	Treatment Arms	Status	Primary Endpoint
NCT05256290	I	Selective 4th gen EGFR TKI	NSCLC with acquired resistance EGFR mutation (eg, C797S) in the absence of concurrent T790M. previous EGFR TKI: mandatory (osimertinib in first line)	BDTX-1535	recruiting	DLT
NCT04862780 (SYMPHONY)	I/II	Selective 4th gen EGFR TKI	NSCLC harboring EGFR T790M and/or C797S mutation previous EGFR TKI: mandatory at least 1 prior EGFR-targeted TKI with activity against the T790M mutation	BLU-945 as monotherapy and BLU-945 in combination with osimertinib	recruiting	DLT
NCT05153408 (HARMONY)	I/II	Selective 4th gen EGFR TKI Chemotherapy	EGFRm NSCLC, EGFR C797X in part 2 previous EGFR TKI: mandatory at least 1 prior 3rd gen EGFR-targeted TKI (osimertinib)	BLU-701 as monotherapy or in combination with either osimertinib or platinum-based chemotherapy	recruiting	MTD safety ORR
NCT02496663	I	Anti-EGFR mAb 3th gen EGFR TKI	EGFR NSCLC previous EGFR TKI: mandatory (either)	Necitumumab + osimertinib	active, not recruiting	MTD safety
NCT03944772 (ORCHARD)	II	MET inhibitor 1st gen EGFR TKI Anti-EGFR mAb ALK TKI RET inhibitor MEK inhibitor Chemotherapy 3th gen EGFR TKI	EGFRm NSCLC previous EGFR TKI: mandatory osimertinib	Biomarker driven: Osimertinib + savolitinib Osimertinib + gefitinib Osimertinib + necitumumab Durvalumab + carboplatin + pemetrexed Osimertinib + alectinib Osimertinib + selpercatinib Durvalumab + carbo/cis-platin + etoposide Osimertinib + carbo/cis-platin + pemetrexed Osimertinib + salumetinib	recruiting	ORR
NCT02609776 (CHRISALYS)	I	Anti EGFR+ MET mAb 3rd gen EGFR TKI Chemotherapy	EGFRm NSCLC naïve or pretreated with TKI previous EGFR TKI: permitted	Amivantamab + Lazertinib + Carboplatin + Pemetrexed	recruiting	DLT, AE, ORR, DOR, clinical benefit rate
NCT04816214 (GEOMETRY-E)	III	MET inhibitor EGFR 3rd gen TKI	EGFR+, T790M-, MET amplification NSCLC previous EGFR TKI: mandatory (either, osimertinib included)	Capmatinib + osimertinib	recruiting	DLT PFS
NCT03940703 (INSIGHT-2)	II	MET inhibitor EGFR 3rd gen TKI	EGFR+, MET amplification NSCLC previous EGFR TKI: mandatory (osimertinib)	Tepotinib + osimertinib	recruiting	DLT ORR
NCT03778229 (SAVANNAH)	II	MET TKI 3rd gen EGFR TKI	EGFRm+/MET+ NSCLC previous EGFR TKI: mandatory (Osimertinib)	Osimertinib + savolitinib	recruiting	ORR
NCT05261399 (SAFFRON)	III	MET inhibitor 3rd gen EGFR TKI Chemotherapy	EGFR NSCLC MET-overexpressed and/or amplified previous EGFR TKI: mandatory (Osimertinib)	Savolitinib + osimertinib vs. platinum-pemetrexed chemotherapy	not yet recruiting	PFS
NCT02099058	I	MET-directed ADC 3rd gen EGFR TKi Anti-PD11st gen EGFR TKI	EGFR+/MET+ NSCLC previous EGFR TKI: mandatory (either)	Telisotuzumab vedotin Telisotuzumab vedotin + osimertinib Telisotuzumab vedotin + erlotinib Telisotuzumab vedotin + nivolumab	recruiting	safety RPTD
NCT04042701	I	HER3-directed ADC anti PD1	HER2+ breast cancer and NSCLC previous EGFR TKI: If EGFR+ NSCLC, mandatory (either Osimertinib included)	Trastuzumab deruxtecan + Pembrolizumab	recruiting	DLT, ORR
NCT03784599 (TRAEMOS)	II	HER2-directed ADC	EGFR+ HER2+ NSCLC previous EGFR TKI: mandatory (either). If 1st or 2nd gen TKI must be T790M negative	TDM1 + osimertinib	recruiting	safety, ORR
NCT05338970	III	HER3-directed ADC	EGFR+ NSCLC previous EGFR TKI: mandatory (third generation TKI)	Patritumab Deruxtecan vs. platinum-pemetrexed based chemotherapy	recruiting	PFS
NCT04619004 (HERTHENA—Lung01)	II	HER3-directed ADC	EGFR+ NSCLC previous EGFR TKI: mandatory (either) + 1 line of platinum based chemotherapy	Patritumab deruxtecan	recruiting	ORR
NCT04676477	I	HER3-directed ADC	EGFR+ NSCLC previous EGFR TKI: mandatory (osimertinib)	Patritumab deruxtecan + osimertinib	recruiting	DLT, afety
NCT03260491	I	HER3-directed ADC	EGFR+ NSCLC previous EGFR TKI: mandatory (either, Osimertinib included)	U3-1402	active, not ecruiting	DLT, ORR
NCT04452877	II	BRAF + MEK inhibitors	BRAF V600E NSCLC previous EGFR TKI: mandatory (either, Osimertinib included)	Dabrafenib + trametinib	recruiting	ORR
NCT04545710	II	CDK4/6 inhibitor 3rd gen EGFR TKI	EGFR+ NSCLC previous EGFR TKI: mandatory (osimertinib)	Abemaciclib + osimertinib	recruiiting	6 months-PFS
NCT03455829	I/II	CDK4/6 inhibitor 3rd gen EGFR TKI	EGFR+ NSCLC previous EGFR TKI: mandatory (osimertinib)	Lerociclib + Osimertinib	active, not recruiting	DLT safety PFS
NCT02729298	I	AXL inhibitor	Solid tumors including EGFR+ NSCLC previous EGFR TKI: mandatory (either, osimertinib included)	TP-0903	active, not recruiting	DLT
NCT03891615	I	PARP inhibitor	EGFR+ NSCLC previous EGFR TKI: mandatory (osimertinib)	Niraparib + osimertinib	recruiting	MTD
NCT04538378	II	PARP inhibitor Anti-PDL1	EGFR+ NSCLC transformed into SCLC in progression to platinum-based chemotherapy previous EGFR TKI: mandatory (either, Osimertinib included)	Niraparib + durvalumab	recruiting	best overall response
NCT04484142 (TROPION-Lung05)	II	HER3-directed ADC	EGFR, ALK, ROS1, NTRK, BRAF, MET exon 14 skipping, or RET positive NSCLC previous EGFR TKI: mandatory (osimertinib included if T790M)	DS-1062a	active, not recruiting	ORR
NCT04765059 (COMPEL)	III	Chemotherapy 3rd gen EGFR TKI	EGFRm+NSCLC in extracranial disease progression previous EGFR TKI: mandatory, (osimertinib)	Platinum/pemetrexed/osimertinib vs. platinum/pemetrexed	recruiting	PFS
NCT04438902	II	Anti-VEGFR TKI 3rd gen EGFR TKI	EGFRm+/T790M NSCLC with gradual progression on osimertinib previous EGFR TKI: mandatory, (osimertinib)	Anlotinib + osimertinib	recruiting	PFS
NCT04405674	II	Anti-PD1 Chemotherapy anti-VEGF mAb	EGFRm+ NSCLC previous EGFR TKI: mandatory, (either, osimertinib if T790M mandatory)	Tislelizumab + carboplatin + nabpaclitaxel followed by tislelizumab + pemetrexed manteinance therapy	recruiting	1 y-PFS rate
NCT02864251	III	Anti-PDL1 Anti-CTLA4 chemotherapy	EGFRm+/T790M- NSCLC previous EGFR TKI: mandatory, (either, osimertinib included)	Nivolumab + platinum + pemetrexed vs. nivolumab + ipilimumab vs. platinum-pemetrexed chemotherapy	active, not recruiting	PFS

Gen—generation; PFS—progression-free survival; mAb—monoclonal antibody; TKI—tyrosine kinase inhibitors; DLT—dose-limiting toxicity; MTD—maximum tolerated dose; ORR—objective response rate.

**Table 2:** Ongoing clinical trials in treatment naïve EGFR-mutated advanced NSCLC.

NCT Identifier	Phase	Drug(s) Class	Population	Treatment Arms	Status	Primary Endpoint
NCT05299125 (AMIGO-1)	II	Anti-EGFR+ MET mAb 3rd gen EGFR TKI Chemotherapy	Advanced NSCLC with common EGFR sensitising mutation	Amivantamab + Lazertinib + carboplatin + pemetrexed	Not yet recruiting	18 months PFS rate
NCT03865511 (MELROSE)	II	3rd gen EGFR TKI	Advanced NSCLC with common EGFR sensitising mutation	osimertinib	recruiting	Genetic profile at disease progression in EGFRm+ compared to baseline
NCT04487080 MARIPOSA	III	Anti-EGFR+ MET mAb 3rd gen EGFR TKI	Advanced NSCLC with common EGFR sensitising mutation	Amivantamb + Lazertinib vs. osimertinib	recruiting	PFS
NCT04248829 LASER301	III	3rd gen EGFR TKI 1st gen EGFR TKI	Advanced NSCLC with common EGFR sensitising mutation	Lazertininb + gefitinib	Active, not recruiting	PFS
NCT04181060 (EA5182)	III	Anti-VEGF mAb 3rd gen EGFR TKI	Advanced NSCLC with EGFR sensitising mutation (uncommon included)	Bevacizumab + osimertinib	recruiting	PFS
NCT03909334	II	Anti-VEGFR2 mAb 3rd gen EGFR TKI	Advanced NSCLC with EGFR sensitising mutation (uncommon included)	Osimertinib + ramucirumab vs. osimertinib	recruiting	PFS
NCT04035486 FLAURA2	III	3rd gen EGFR TKI chemotherapy	Advanced NSCLC with EGFR sensitising mutation	Osimertinib + platinum-pemetrexed chemotherapy vs. osimertinib	Active, not recruiting	PFS
NCT03567642	I	3rd gen EGFR TKI chemotherapy	Advanced EGFR+ NSCLC with concurrent RB1 and TP53 Alterations	Platinum-etoposide + osimertinib	recruiting	MTD
NCT03392246	II	MEK inhibitor 3rd gen EGFR TKI	Advanced NSCLC with EGFR sensitising mutation	Osimertinib + salumetinib	recruiting	Best objective response
NCT04695925	III	3rd gen EGFR TKI chemotherapy	Advanced EGFR+ NSCLC with concurrent TP53 mutation	Osimertinib vs. Osimertinib + carboplatin + pemetrexed	Not yet recruiting	PFS
NCT02971501	II	Anti-VEGF mAb 3rd gen EGFR TKI	Advanced NSCLC with EGFR sensitising mutation with brain metastasis	Bevacizumab + osimertinib	Active, not recruiting	PFS
NCT02954523	I/II	BCR/AbL inhibitor 3rd gen EGFR TKI	Advanced NSCLC with EGFR sensitising mutation (uncommon included)	Dasatinib + Osimertinib	Active, not recruiting	Safety
NCT03122717	I/II	3rd gen EGFR TKI 1st gen EGFR TKI	Advanced NSCLC with EGFR sensitising mutation	Osimertinib + gefitinib	Active, not recruiting	Number of patients completing combination therapy for 6 × 28 day cycles

Gen—generation; PFS—progression-free survival; mAb—monoclonal antibody; TKI—tyrosine kinase inhibitors.
